# New Drugs, Appliances, and Things Medical

**Published:** 1889-11-02

**Authors:** 


### NEW DRUGS, APPLIANCES AND THINGS MEDICAL.

[All preparations, appliances, novelties, etc., of which a notice is desired, should be sent to The Editor, The Lodge, Porchester Square, W.]

### REVOLUTIONS IN TEA—SANTHA.

There is a new tea. A tea without the tannin, or, at any rate, one in which that constituent is made harmless by combination. Henceforth anyone may swallow his sixteen cups of "Santha," as it is named, and emulate Dr. Johnson in his tea-drinking feats without fear of consequences.

These are days of change indeed. The discovery of steam as a motive power simply revolutionised the whole condition of society. There are not wanting signs, however, that steam, so far as machinery is concerned, has had its day, and will soon follow the stage coach, the old three-decker man-o'-war, and many another once familiar figure of the past. But customs of purely social origin change less quickly than those dependent on scientific discoveries. They are slow of coming and loth to take their departure, and some, indeed, seem as if they intend to stay on for ever. Among the latter may be mentioned three importations into our country that have had no small influence upon our history and social developments—namely, the potato, tobacco, and tea. The introduction of the potato had much to do with the increase of population, and hence of national prosperity, by providing an easily-cultivated source of food. Of tobacco less can be said as an active factor in history. It is an article of luxury, yet who, looking at its large consumption by the biggest brained nations, can doubt that the weed has had its influence in shaping the destinies of mankind? Our present poet-laureate is an inveterate smoker; almost every German smokes, and the use of tobacco is well-nigh universal in the realms of Bohemia, whence the army of artists, journalists, poets, and novelists is drawn. Of the third article mentioned, tea, also one of luxury, there can be no doubt that it has helped to work a revolution in our midst. All through the middle ages the consumption of beer and spirits was very large; indeed, no one without an iron constitution could have lived through those piping times. All the hard fighting and the harder living have no doubt served a useful purpose in evolution, for they have created a robust, hardy, and adventurous race of men. However, our immediate subject is tea. For a long time after its first introduction the high price of tea made it a luxury confined to the few, and our great-grandmothers drank the costly beverage out of the tiniest of cups. The Government duty was large, six shillings per pound. It is now the same number of pence, and tea has grown cheaper and cheaper as the duty has been lowered, so that good leaf is brought within reach of the peasant. Of late years the China trade has been seriously affected by East India competition. The Chinese felt so sure of their market that they became careless of their methods of preparation, and flooded the English market with any sort of rubbish. Indian tea, on the other hand, is carefully made so as to preserve all its delicate aromatic properties. It is not surprising, therefore, that the latest report of the tea market points out the facts that our consumption of Indian and Ceylon teas is rapidly progressive, and that our trade with those dependencies during 1888 actually exceeds in amount the trade with China.

The following account of the action and properties of this important article is given by Mr. Henry Burdett in his work* "Helps to Health": "Tea consists, as everyone knows, of the leaves of a plant grown in China, and is divided into two chief kinds—black and green tea—from the different manner of their preparation. Its chief constituents are tannin or tannic acid—a substance with a very astringent taste—a volatile oil to which it owes its aroma, and a special substance known as theine. It is prepared by pouring boiling water on it, and allowing it to stand a short time. If it stands too long, as many poor people let it, simmering on the hob all through the day, it loses of necessity its volatile oil which gives it its agreeable flavour, and takes up more of the tannin and bitter, disagreeable matter. The same occurs if it is boiled. In England it is customary to add milk and sugar to it; in other countries other things are added. The best tea contains a good proportion of small, young leaves. It produces two distinct sets of effects. 1. It stimulates the brain, dispelling drowsiness, and is not followed by torpor, as after the use of alcohol. 2. It lessens tissue waste, and consequently the desire for food and the sense of fatigue from overwork. It is thus of use in those especially who wish to do hard mental work at night, also in those who are exposed to much fatigue, especially under extremes of temperature. The excessive use of tea by women of the lower class is a fertile source of indigestion, probably from the bad manner of its preparation, as it is allowed to stand for an indefinite time, and then contains a large amount of tannin and substances that act injuriously on the coats of the stomach. Women also allow it to take the place of more solid and essential nutriment, and the only meal—morning, noon, and night—of many of them is tea, with bread and butter."

"Santha" appears to be an extract of tea, in which the tannin is combined to make an insoluble compound, by which means it is rendered inert. The preparation is made on scientific principles, and should it prove a success, as there is every prospect of its doing, there will be many people willing to give it a trial. There are tea-drinkers of all sorts, ranks, and conditions: the pale student burning the midnight oil, the overworked doctor called out at night, the wearied nurse, the huge army of dyspeptics, brain workers, toilers in the field, washerwomen, and women of fashion.

* Page 92.

